# Cancer patient experience of telephone clinics implemented in light of COVID-19

**DOI:** 10.1177/1078155221990101

**Published:** 2021-01-27

**Authors:** Melanie Dalby, Alison Hill, Shereen Nabhani-Gebara

**Affiliations:** 1 Barts Health NHS Trust, London, UK; 2 Department of Pharmacy, Kingston University, Kingston upon Thames, UK

**Keywords:** Telephone clinics, patient experience, COVID-19, cancer

## Abstract

**Introduction:**

Due to the pandemic of COVID-19 a number of National Health Service (NHS) Trusts in the UK adopted telephone consultations for patients who were shielding. As the pandemic continues to affect these services an evaluation was conducted to determine whether telephone consultations implemented during the pandemic should be maintained long term. The objective was to evaluate this new service and to understand patient experience.

**Methods:**

This study was conducted via a telephone survey. Staff working in the Macmillan centres across the Trust called patients to survey them about their experience of telephone consultations. Data were collected 23/06/20 – 17/07/20. A mix of eight open and closed questions were asked. Data were collected on an Excel spreadsheet and patient identifiable information was anonymised.

**Results:**

55 patients accepted to participate in this study. Out of 55, 39 patients rated the phone consultation they had as either 4 or 5 out of 5. When asked if they would like to continue with phone clinics 33 said they would. The majority of consultations were conducted by doctors (43/55). Patients commented they had received great support from their healthcare professionals and they felt that phone consultations were safer in the current climate. Three of the patients felt the calls were rushed and others found it difficult to discuss pain management, sides effects and post-surgery issues.

**Conclusions:**

This evaluation provides a brief snapshot of the experience cancer patients are having with phone clinics. A re-evaluation will take place once video consultations are implemented.

## Introduction

Healthcare systems across the world had to rapidly implement innovative changes to continue safe, effective care of their patients as COVID-19 started to spread at the beginning of 2020.^1^ The World Health Organisation (WHO) declared the COVID-19 virus a pandemic on 11th March 2020.^2^ One of the innovative methods that healthcare systems chose was to introduce virtual care. There are several different terms used for virtual care. Shaw et al describe it as any remote interaction between a patient and a healthcare provider using technology.^3^ Miah et al named it telemedicine^4^ and Tashkandi et al have coined the application of telemedicine in oncology as teleoncology.^5^

There is plenty of evidence for the benefits of virtual care which have shown successful application of technology in palliative care, survivorship care and symptom management.^5–8^ Benefits for the patient include reduced travel times,^9^ reduced costs associated with travelling, reduced waiting times and reduced impact of travel on symptoms.^10^ They may feel more comfortable receiving their consultation in the comfort of their own home.^11^ The reduction in travel provides an added benefit to the environment by reducing the carbon footprint.^4,12^ One study in Saudi Arabia looked at how oncologists managed during the implementation of virtual management of patients during the pandemic.^5^ It showed that oncologists have a high level of awareness of virtual technology and 46% (n = 222) said they definitely preferred managing some cases virtually with only 36% saying they will continue virtual management after the pandemic.^5^

Of course, virtual care is not a new concept as many NHS Trusts in the UK successfully use telephone clinics^13^ not just in cancer. For example virtual clinics have been shown to be successful in routine follow-up after general surgery.^14^ This resulted in a reduction in unnecessary appointments allowing for more timely access for newly referred patients.^14^ Video consulting has also been successful within primary care.^15^ These examples are likely to have been driven by the NHS Long Term Plan which recommends the uptake of digital innovations to improve patient care and health management.^16^

The purpose of this study was to determine the experience of cancer patients whose outpatient appointments changed from face-to-face to telephone during the pandemic. Prior to the pandemic most of the outpatient clinics were conducted in person with only a few follow-up clinics taking place via the telephone. Like so many other healthcare systems a new approach was required at the start of the COVID-19 pandemic. In a short period of time outpatient staff, medical, clinical and haematology teams worked hard to make the change to telephone consultations. A review of the experience of patients is required to determine whether this model would be sustainable going forward and would help in the implementation of video consultations.

## Method

Purposive sampling was used to select the patient sample. Initially a report was pulled from the electronic patient record system. The criteria for this report was patients who had a cancer diagnosis and had been admitted for a day case in the last six months. The report was exported to excel, and the author selected patients who had had a telephone consultation since 23rd March. These patients were transferred to a second list.

A telephone survey guide was prepared with a data collection tool. Staff working in the four Macmillan centres across the Trust were trained on how to use both the guide and the data collection tool. The staff called up patients by telephone on the second list between 23rd June 2020 – 17th July 2020. The staff divided up the list between them and called patients until the selected sample size was reached and there was a mix of patients with different cancers and from different hospital sites. Eight questions were asked using a mix of open and closed questions. The requested sample size was 60 patients. This would provide an initial insight to patient experience without using excessive amounts of staff resourcing. The Trust is made up of 4 main hospitals and 1 community site with a variety of cancer services spread throughout. A suggested sample of 60 was chosen as it was thought that this would provide a suitable mix of patients with different cancers who had had a telephone clinic from the different sites. All patient data was anonymised.

Descriptive statistics was used to analyse the quantitative data. As the telephone surveys were not recorded the qualitative data could not be analysed verbatim, however common themes were drawn from what the interviewers documented during their discussions with the patients. As this study was deemed a service evaluation ethics approval was not required.

## Results

A total of 55 patients took part in a telephone survey. The requested sample size was not reached as some patients on the list were uncontactable, it was deemed inappropriate to call them due to recent medical events or on further investigation had not had a cancer telephone clinic. At this point the main author determined that there were sufficient data from a mix of patients with different cancers who had had a telephone clinic originating from one of more of the different hospital sites and that data saturation was reached. The demographics of the patients are listed in Table 1.

**Table 1.** Patient demographics.

	Total
Age	
21–40	10
41–60	17
61–80	22
>80	6
Gender	
Male	27
Female	28
Ethnicity	
Asian – Asian British – Indian	3
Asian – Asian British – Bangladeshi	1
Asian or Asian other background	3
Black – Black British Caribbean	4
Black – Black British African	10
White British	21
White – Any Other White Background	5
Other/unknown	8
Cancer type	
Breast	18
Lung	2
Prostate	9
Gynae	4
Melanoma	2
Myeloma	2
CRC/GI	3
Bladder	1
Renal	5
Testicular	3
Hodgkin Lymphoma	2
Haem other	4

The majority of telephone consultations were described as follow-up consultations (Table 2). Most consultations were conducted by doctors (43/55) but some were conducted by nurses or conducted by both nurse and doctor. Out of 55 patients, 39 patients rated the telephone consultation they had as either 4 or 5 out of 5 (1 – very dissatisfied and 5 – very satisfied). When asked if they would like to continue with phone clinics 33 said they would, 18 said no, 1 patient was unsure and 3 would like to alternate telephone with face-2-face consultations.

**Table 2.** Quantitative data obtained during the telephone surveys.

Question	Number of respondents
Purpose of call	
Follow up	40
Breaking bad news	0
Results	9
Treatment option discussion	3
To determine fitness to continue with treatment	3
Who was the call with?	
Doctor	43
Nurse	4
Both	8
Rating of consultation out of 5	
1	2
2	4
3	10
4	13
5	26
Would you like to continue with virtual clinics?	
Yes	33
No	18
Unsure	1
Yes but only alternate appointments	3

Qualitative data capture was documented by the staff undertaking the phone survey. This was either documented as the patient was talking or written down after the telephone call had taken placed. The data was categorised into six themes (Table 3). The fourth theme is split into two sub-themes.

**Table 3.** Themes from the qualitative data with corresponding descriptions.

Theme	Description	
Rushed	On the phone it felt rushed, as [the patient] could not discuss things at length.	
	The phone calls feel rushed, and [the patient] tended to forget important information due to this.[The patient] felt rushed, lots of questions unanswered.	
Face to face	[The patient is] quite an anxious person and would prefer the face to face appointments.	
	[The patient] wanted face to face contact as she felt that her condition required eyes to see.	
	[The patient feels] much more comfortable when it is face to face.	
	Prefers in person. Telephone less personal.	
	Prefers face to face. Telephone useful if unwell.	
	[The patient] did miss face to face contact.	
Difficult to assess	[The patient] has found it hard due to her treatment side effects which are causing problems in the hands and feet and she felt she could not explain these side effects well over the phone.	
	It was her first appointment after her surgery and she really needed the doctor to see the breast surgery site. She did not know whether the healing was ok.	
	He would like someone to see his left ankle and see if the screw can be taken out and because they couldn't see it, it was hard to understand what to do next. They could not examine him over the phone and he really hopes that face to face appointments are available soon.	
	[The patient] found it a real struggle and felt that he really needed to see someone so he can talk face to face. The pain has been unmanageable and phone calls have not helped him too much.	
	Symptoms of recurrence are very difficult to assess over the phone, so the appointment was pointless.	
	Just needed to be examined by the doctor.	
	[The patient] would not want to miss out on a physical exam where necessary.	
	It is difficult to explain to the doctor the side effects and how the healing journey has been. [The patient] feels very tender and cannot explain her symptoms very well so would prefer to have someone feel and check.	
Communication	Positive communication	Fully informative phone calls by the oncologist and Urology team.
		[The patient] had a bleed and was very scared, she called her consultant and was called back straight away and given a face to face meeting the following week.
		The doctor gave me time to understand. Clear information, answered all my questions.
		Very good - she spoke to my daughter as my English is not good.
		[The patient] felt like it was easy to discuss anything over the phone.
		[The patient] found the phone conversations quite hard … Not being able to discuss her treatment plan at length is something she found extremely difficult.
	Barriers to communication	[When the patient] has a face to face appointment, she was able to get an interpreter whereas on the phone her son has had to translate. She felt that even through translation she was unable to get her side effects across to the doctor.
		Was hard to understand at some times.
Benefits	Saves time with virtual clinic. Finds coming up to hospital very stressful.	
	Saved travelling and sitting in waiting room for very short appointment.	
	Liked that she did not have to leave her house.	
	Less stress knowing I did not have to leave home.	
	No travel and no virus risk.	
	No travel or childcare needed	
Compassionate care	[The patient’s] CNS has been able to help him with any questions he has had and supported him throughout.	
	[The patient’s] nurse has been very supportive, and attentive and also giving advice.The phone calls picked her up when she has not been feeling great.	
	Consultant took time to explain. Answered all my questions, felt listened too.	
	Still felt friendly as has known consultant for long time.	
	The doctor was calming. Very thorough and the top of his game.	
	The doctor looked after me very well. The doctor was a good listener, felt he cared.	
	[The patient] knows that he can call his CNS whenever he needs … feel supported in these difficult times.	

## Discussion

The pandemic of COVID-19 has been a difficult time not only for patients^17^ with cancer and their families but also staff working in cancer in NHS hospitals.^18^ It is essential when introducing new ways of working that an evaluation is conducted.^19^ The purpose of this study was to evaluate the wider implementation of telephone clinics within our cancer services across the Trust.

This study provided a brief snapshot of the experience of patients who received a telephone consultation instead of their usual face to face consultation during the first wave of the pandemic. Many of the calls were follow up calls. There were no consultations where bad news was given. Rimmer^20^ explains the difficulties of breaking bad news over the phone and provides advice. It might be that none of the patients that were selected through our purposive sampling were in a situation where bad news had occurred or that our consulting teams were still using face to face appointments for this type of consultation. Follow up consultations do make up a lot of the appointments normally therefore this figure is in keeping with normal practice.

Most of the patients were reviewed by doctors alone.^21^ It was unclear why some patients were seen by both a nurse and a doctor. This could have been due to patient complexity or due to a more MDT approach to consultations in some tumour groups compared to others.

As shown 39 patients rated the telephone consultation as either 4 or 5 out of 5 and 33 would like to continue with them going forward. This is a very rudimentary scoring system but it suggests that the majority of our patients had a positive experience. Certainly, from the qualitative data they were understanding of the reasons as to why telephone clinics were introduced. In a study by Beaver et al^11^ comparing hospital and telephone follow-up after treatment for breast cancer they found that those in the telephone group reported greater satisfaction with the information they received and found the support more helpful.

The qualitative data was characterised into six themes (Table 3). Even though there were only three patients who described the calls as rushed it was important to note the impact and experience that these patients would have had consequently. The telephone consultations would have been new to a lot of staff and therefore it would have taken a few consultations before staff themselves felt comfortable.

The phrase ‘face to face’ was mentioned numerous times and there clearly were some patients who preferred this option over the telephone option. Most of the time it seemed to be more a personal preference however there were examples where the patient felt they needed to be physically seen and an anxious patient who may have felt calmer seeing someone in person. This leads onto the third theme whereby certain patients identified the issue with trying to describe their condition where perhaps a physical assessment was warranted. Having a set of inclusion and exclusion criteria for telephone consultations would be beneficial in this circumstance as then for example the breast surgery patient would perhaps fall into the exclusion criteria and would be automatically contacted for a face to face appointment to review wound care and healing.

There is a mix of opinions in terms of the overall communication. Some patients described their telephone conversation as very informative whereas others had a harder time. One patient did not like the fact that her son had to translate rather than the doctor organising a translator. The patient felt this hindered describing of their side effects. Another patient was happy for their daughter to translate and speak to the doctor. In these circumstances it may have been useful to speak to the son and daughter to find what their experience was. Reasons why a patient may have found it difficult could be because they were hard of hearing or the telephone line was not clear.

The majority of the patients saw the benefits of the telephone consultations all of which complement the literature described previously.^9–11^ These benefits are likely to support the maintenance and sustainability of virtual consultations going forward. The last theme of compassionate care details comments where staff made the patient feel at ease and gave them the time they needed. These comments show that telephone consultations can work successfully, and it is worth evaluating and improving new services to ensure all cancer patients receive the same experience as these patients.

With a second wave now upon us it is likely that telephone clinics will need to continue as an important method for cancer care delivery.^22^ A small number of our patients suggested the idea of alternating face to face consultations with telephone consultations. This has also been suggested by Parish et al.^21^ who reported that this alternation would provide patients with more scrutiny. This would benefit those patients who are suffering with significant side effects.

As the pandemic of COVID-19 continues the method in which cancer outpatient clinics are conducted needs to be flexible and adaptable. As telephone consultations have been implemented more widely, video consultations have also started. Video consultations do require more technology^9,23^ than telephone consultations but will offer added benefits.^12,24,25^ For example video consultations may benefit our patient suffering with palmar-plantar erythrodysesthesia as she will be able to show her clinician the effects as well as describe them.

This work has been fed back to the Trust cancer directorate. A further service evaluation will be conducted in the new year to include those patients who have received a video consultation. It would also be beneficial to interview clinicians to find what their experience has been as well as any family members who also took part in the consultations. A further amendment to the method would be to record the telephone survey to allow for verbatim transcription and full thematic analysis. A limitation from this study that will be present in the future study is that some patients may be reluctant to be completely honest as the feedback method is not anonymous. This study provides baseline data and learning which can be built on to further improve our outpatient services to cancer patients.
